# Relationships among Smoking Habits, Airflow Limitations, and Metabolic Abnormalities in School Workers

**DOI:** 10.1371/journal.pone.0081145

**Published:** 2013-11-29

**Authors:** Masafumi Horie, Satoshi Noguchi, Wakae Tanaka, Yasushi Goto, Hisanao Yoshihara, Masaki Kawakami, Masaru Suzuki, Yoshio Sakamoto

**Affiliations:** 1 Dept of Respiratory Medicine, Graduate School of Medicine, University of Tokyo, Bunkyoku, Tokyo, Japan; 2 Dept of Allergy and Respiratory Medicine, Teikyo University, Itabashiku, Tokyo, Japan; 3 Dept of Respiratory Medicine, Kanto Central Hospital, Setagayaku, Tokyo, Japan; Research Center Borstel, Germany

## Abstract

**Background:**

Chronic obstructive pulmonary disease is caused mainly by habitual smoking and is common among elderly individuals. It involves not only airflow limitation but also metabolic disorders, leading to increased cardiovascular morbidity and mortality.

**Objective:**

We evaluated relationships among smoking habits, airflow limitation, and metabolic abnormalities.

**Methods:**

Between 2001 and 2008, 15,324 school workers (9700 males, 5624 females; age: ≥30 years) underwent medical checkups, including blood tests and spirometry. They also responded to a questionnaire on smoking habits and medical history.

**Results:**

Airflow limitation was more prevalent in current smokers than in ex-smokers and never-smokers in men and women. The frequency of hypertriglyceridemia was higher in current smokers in all age groups, and those of low high-density-lipoprotein cholesterolemia and diabetes mellitus were higher in current smokers in age groups ≥ 40 s in men, but not in women. There were significant differences in the frequencies of metabolic abnormalities between subjects with airflow limitations and those without in women, but not in men. Smoking index was an independent factor associated with increased frequencies of hypertriglyceridemia (OR 1.015; 95% CI: 1.012–1.018; p<0.0001) and low high-density-lipoprotein cholesterolemia (1.013; 1.010–1.016; p<0.0001) in men. Length of smoking cessation was an independent factor associated with a decreased frequency of hypertriglyceridemia (0.984; 0.975–0.994; p = 0.007).

**Conclusions:**

Habitual smoking causes high incidences of airflow limitation and metabolic abnormalities. Women, but not men, with airflow limitation had higher frequencies of metabolic abnormalities.

## Introduction

Chronic obstructive pulmonary disease (COPD) is an important and growing cause of morbidity worldwide. The Burden of Lung Disease Study [1] reported that the worldwide prevalence of COPD at stage II or higher was 10.1%. In Japan, the Nippon COPD Epidemiology (NICE) study also showed that the prevalence of airflow limitation was 10.9%, higher than that previously reported and suggesting a high degree of under-recognition of COPD [2].

Cigarette smoking is the major cause of COPD, and encouraging smoking cessation is essential for the management of COPD because it can reduce the rate of decline in forced expiratory volume in 1 s (FEV_1_) [3]. Early diagnosis of COPD and early intervention for smoking cessation is important for preventing loss of pulmonary function, although only 15–20% of smokers are ever diagnosed with COPD [4].

Airflow limitation in COPD is caused by inflammation of small airways and destruction of the lung parenchyma. Besides airflow limitation, systemic inflammation in COPD causes various extrapulmonary manifestations, and comorbidities occur frequently, affecting the natural history of the disease [5]. Several studies have demonstrated that COPD is an independent risk factor for cardiac mortality [6], [7], and people with impaired lung function have a higher probability of death from complications of atherosclerotic vascular disease [8]. Furthermore, a positive independent relationship between lung function impairment and metabolic syndrome has been reported [9].

Regarding metabolic syndrome, a meta-analysis showed that smokers had dysregulated lipid metabolism, including significantly higher serum concentrations of total cholesterol (TC), triglycerides (TG), very-low-density lipoprotein cholesterol (VLDL-C), and low-density-lipoprotein cholesterol (LDL-C), and lower serum concentrations of high-density-lipoprotein cholesterol (HDL-C) than never-smokers [10]. A Japanese cross-sectional and longitudinal cohort study suggested that in men, the greatest difference in TG levels between smokers and non-smokers was seen in middle age, but in women, the greatest difference was seen after middle age, indicating age- and gender-dependent effects of smoking on serum lipid levels [11]. Thus, the relationship between habitual smoking and lipid metabolism is becoming clearer, but the effect of smoking cessation on these markers remains unclear. A previous meta-analysis showed that smoking cessation resulted in elevated HDL-C levels, but no significant reduction in TC, LDL-C, or TG [10]. Gerace et al. [12] also reported that ex-smokers had an adjusted increase of 2.4 mg/dL HDL-C but no decrease in TC or LDL-C levels, compared to smokers.

Therefore, we performed a cross-sectional study to evaluate not only the relationships among smoking habit, airflow limitation, and metabolic abnormalities, but also the impact of smoking cessation on these factors.

## Methods

### Study Design and Participants

Between 2001 and 2008, a total of 29,469 school workers underwent medical checkups at Kanto Central Hospital, a key hospital located in Tokyo serving all public school workers in the Kanto area. Subjects who underwent both spirometry and blood tests were included in this study; subjects lacking data for either were excluded. Because medical checkups were performed annually, some subjects underwent examinations twice or more during the period. In such cases, the oldest data were used for analysis, and other data were excluded. Finally, 15,324 subjects (9700 males, 5624 females; age ≥ 30 years, mostly teachers) were enrolled.

On the night before the day of their examinations the subjects stayed at the hospital and ate the same type of dinner. On the next morning, their blood pressure, height, weight, and body mass index (BMI) were measured. Venous blood was obtained before breakfast. Serum TC, LDL-C, HDL-C, fasting blood sugar (FBS), 2 h oral glucose tolerance test (OGTT) glucose, HbA1c, uric acid (UA), albumin, and high-sensitivity C-reactive protein (CRP) were measured. Each subject was asked to answer a questionnaire on history of cigarette smoking, alcohol use, diseases, medications, and physician-diagnosed bronchial asthma (BA). Pulmonary function tests were performed between 10 and 11 am. TG levels over 150 mg/dL or requiring hypolipidemic drug therapy were defined as hypertriglyceridemia. HDL-C levels under 40 mg/dL or requiring hypolipidemic drug therapy were defined as low HDL cholesterolemia. LDL-C levels over 140 mg/dL or requiring hypolipidemic drug therapy were defined as high LDL cholesterolemia. FBS levels over 126 mg/dL, 2 h OGTT glucose levels over 200 mg/dL, HbA1c levels (NGSP) over 6.5%, or requiring a hypoglycemic agent were defined as diabetes mellitus (DM). Uric acid levels over 7.0 mg/dL or requiring hypouricemic agents were defined as hyperuricemia (HU).

Because measurement of LDL-C was not started until 2001 and the stay at the hospital was abandoned after 2008, data between 2001 and 2008 were collected and analyzed.

The present study was approved by the Ethics Committee of Kanto Central Hospital. The decision of the committee was that it was unnecessary to ask each participant to provide written informed consent, unless the participant refused to allow us to use the participant's own data for the statistical analysis after reading the official statement by both The Japan Society of Ningen-Dock (Medical Checkup) and The Japan Hospital Association asking for understanding and cooperation for the use of clinical data for the purpose of promoting the improvement of nationwide public health. To date, nobody has refused.

### Pulmonary Function Tests

Spirometry was performed by trained technicians according to the American Thoracic Society recommendations [13] using an auto-spirometer (Spiroanalyzer ST-200; Fukuda Denshi Co., Ltd., Tokyo, Japan). Vital capacity, forced vital capacity (FVC), FEV_1_, and the FEV_1_/FVC ratio were calculated. Reference values for predicted FEV_1_ were calculated using the equation prepared by the Japanese Society of Respiratory Diseases [14]. The best FEV_1_ and FVC values were used for the analysis according to the recommendation of the American Thoracic Society and the European Respiratory Society [13], [15]. Among the subjects with FEV_1_/FVC ratio < 0.7, mild, moderate, severe, or very severe airflow limitation was defined by the percentage of predicted FEV_1_ ≥ 80%, 50–80%, 30–50%, or < 30%, respectively, by reference to the Global Initiative for Chronic Obstructive Lung Disease (GOLD) [16]. All subjects underwent spirometry without inhalation of a β_2_-agonist.

### Statistical Analysis

Statistical analyses were performed using the JMP software (ver. 9; SAS Institute Japan Ltd., Tokyo, Japan). Pearson's chi-squared test was used for the comparison of two groups with a dichotomous dependent variable, and Student's t-test was used to compare the means of two samples. Analysis of variance (ANOVA) was used for multiple comparisons. When ANOVA indicated significant differences between the groups, a Tukey-Kramer test was used. Logistic regression analysis was used to estimate odds ratios (OR) for each of the independent variables in the model. The statistical significance level was set at p<0.05.

## Results

### Smoking habit in men and women

The subjects were divided into four age groups (30 s, 40 s, 50 s, and over 60) and three further groups (current smokers, ex-smokers, and never-smokers). Smoking rate, smoking index (SI; packs × year), and years after cessation were surveyed for each age group. The proportions of current smokers in men and women were 22.9% and 4.4%, respectively. Those of ex- and never-smokers in men and women were 34.8% and 4.7%, and 42.3% and 90.9%, respectively (Table 1).

**Table 1:** Smoking habits in men and women.

Age group	CS		Ex S			NS	Total
	n (%)	SI	n (%)	SI	Smoking cessation (yr)	n (%)	n
**Men**							
30 s	222	15.0	129	7.5	5.0	342	693
	(32.0)	(9.0–19.3)	(18.6)	(3.0–13.0)	(1.0–9.8)	(49.4)	
40 s	677	22.5	762	10.0	10.0	1149	2588
	(26.2)	(16.1–38.0)	(29.4)	(5.0–20.0)	(4.0–18.0)	(44.4)	
50 s	906	32.0	1531	15.0	15.0	1694	4131
	(21.9)	(23.4–29.0)	(37.1)	(7.0–27.0)	(6.0–24.0)	(41.0)	
over 60 s	418	38.0	955	20.0	20.0	915	2288
	(18.3)	(25.4–45.0)	(41.8)	(10.0–30.0)	(8.0–27.0)	(40.0)	
Total	2223		3377			4100	9700
	(22.9)		(34.8)			(42.3)	
							
**Women**							
30 s	28	6.8	15	3.0	3.0	197	2410
	(11.7)	(2.6–10.9)	(6.3)	(2.5–10.6)	(1.5–8.5)	(82.0)	
40 s	57	10.0	70	2.5	8.0	996	1123
	(5.1)	(6.0–16.3)	(6.2)	(1.4–5.5)	(2.0–19.5)	(88.7)	
50 s	118	10.0	123	4.8	10.0	2616	2857
	(4.1)	(6.2–22.3)	(4.3)	(1.5–9.0)	(3.0–20.0)	(91.6)	
over 60 s	44	15.0	55	7.5	10.0	1305	1404
	(3.3)	(4.4–24.4)	(3.9)	(2.5–15.0)	(3.0–15.0)	(93.0)	
Total	247		263			5114	5624
	(4.4)		(4.7)			(90.9)	
							
**Overall**	2470		3640			9214	15 324
	(16.1)		(23.8)			(60.1)	

*Definition of abbreviations*: SI  =  smoking index; CS  =  current smoker; Ex S  =  ex-smoker; NS  =  never-smoker. Data are expressed as medians (interquartile range).

### Prevalence of airflow limitation in men and women

Airflow limitation (FEV_1_/FVC ratio <0.7) without physician-diagnosed asthma was demonstrated in 867 of 9457 (9.2%) men, and 168 of 5,447 (3.1%) women. The prevalence of overall airflow limitation, and moderate-to-very-severe airflow limitation was surveyed for each age group. The frequency of airflow limitation increased in proportion to age and smoking status: 14.9% in men and 8.7% in women in current smokers, 10.5% in men and 2.0% in women in ex-smokers, and 5.0% in men and 2.9% in women in never-smokers (Table 2). In men, the frequencies of moderate-to-very-severe airflow limitation were 5.4% in current smokers, 3.5% in ex-smokers, and 1.0% in never-smokers. In women, the numbers were 2.9% in current smokers, 0.4% in ex-smokers, and 0.7% in never-smokers.

**Table 2:** Frequencies of overall airflow limitation, and moderate to very severe airflow limitation in men and women.

Age Group	FEV_1_/FVC ratio < 0.7			FEV_1_/FVC ratio < 0.7 and % pFEV_1_ < 80%		
	CS (%)	Ex S (%)	NS (%)	CS (%)	Ex S (%)	NS (%)
**Men**						
30 s	1.4	3.9	2.8	0.0	1.6	0.3
40 s	6.2	4.5	3.2^*^	1.7	0.7	0.7
50 s	19.1	8.5^*^	5.4^*†^	6.7	2.9^*^	1.1^*†^
over 60 s	26.6	19.2^*^	7.4^*†^	11.2	7.0	1.5^*†^
Total	14.9	10.5^*^	5.0^*†^	5.4	3.5^*^	1.0^*†^
**Women**						
30 s	0.0	0.0	1.1	0.0	0.0	0.5
40 s	5.6	1.5	1.6	1.9	0.0	0.1
50 s	10.2	2.6	2.4^*^	2.6	0.0	0.6
over 60 s	13.6	1.9	5.0	6.8	1.9	1.3
Total	8.7	2.0^*^	2.9^*^	2.9	0.4	0.7^*^
**Overall**	14.3	9.9^*^	3.8^*†^	5.1	3.3^*^	0.8^*†^

*Definition of abbreviations*: FEV_1_  =  forced expiratory volume in 1 second; % pFEV_1_  =  FEV_1_/predicted FEV_1_×100%; FVC  =  forced vital capacity; CS  =  current smoker; Ex S  =  ex-smoker; NS  =  never-smoker; ^*^, significantly different from CS (p<0.01); ^†^, significantly different from Ex S (p<0.01).

### Relationship between smoking habit and metabolic abnormalities in men and women

We analyzed the relationship between smoking habit and metabolic abnormalities (Table 3). In men, the frequencies of hypertriglyceridemia in current smokers were higher than in ex-smokers or never-smokers in all age groups. The frequencies of low HDL cholesterolemia were higher in current smokers than in ex-smokers or never-smokers in the 40 s and 50 s age groups. On the other hand, there was no significant difference in the frequency of high LDL cholesterolemia in any age group. DM was more frequent in current smokers than in ex-smokers or never-smokers in the 40 s, 50 s, and over 60 s age groups. In women, there was no significant difference in any parameter among current smokers, ex-smokers, and never-smokers in any age group.

**Table 3:** Relationship between smoking and metabolic abnormalities in men and women.

Age group	Smoking status	Men						Women					
		BMI	h-TG	l-HDL	h-LDL	DM	HU	BMI	h-TG	l-HDL	h-LDL	DM	HU
		(kg/m^2^)	(%)	(%)	(%)	(%)	(%)	(kg/m^2^)	(%)	(%)	(%)	(%)	(%)
30 s	CS	24.5	37.4	11.3	27.5	3.6	39.2	19.9	10.7	0.0	3.6	3.6	0.0
	Ex S	23.9	27.9	4.7	24.8	3.1	38.8	20.3	13.3	0.0	13.3	0.0	0.0
	NS	24.2	24.9^*^	8.5	27.2	3.2	33.0	21.7	5.1	1.0	11.7	0.5	0.5
													
40 s	CS	24.5	40.3	13.0	36.3	11.7	31.8	22.3	8.8	3.5	14.0	7.0	1.8
	Ex S	24.5	33.3^*^	10.5	42.3	9.1	36.5	22.6	5.7	1.4	28.6	2.9	1.4
	NS	24.4	29.1^*^	8.7^*^	37.5	7.6^*^	30.2^†^	22.5	7.3	2.2	21.0	3.5	1.6
													
50 s	CS	24.0	40.1	19.0	35.9	23.8	24.3	22.9	18.4	4.2	43.2	7.6	1.7
	Ex S	24.4^*^	34.3^*^	12.3^*^	41.9	15.9^*^	31.9^*^	22.9	23.6	11.4	49.6	5.7	3.3
	NS	23.9^†^	26.7^*†^	11.6^*^	39.0	14.3^*^	25.6^†^	22.9	17.7	7.2	48.7	8.6	2.8
													
over 60 s	CS	23.6	36.4	16.3	35.2	25.4	30.1	22.3	15.9	6.8	40.9	11.4	4.6
	Ex S	23.8	29.5	14.5	42.0	21.3	30.1	23.6	29.1	18.2	58.2	10.9	3.6
	NS	23.6	22.4^†^	11.6	40.0	18.8^*^	24.2^†^	22.8	21.8	13.1	54.0	11.7	3.2

*Definition of abbreviations*: BMI  =  body mass index; h-TG  =  hypertriglycemia; l-HDL  =  low high-density lipoprotein cholesterolemia; h-LDL  =  high low-density lipoprotein cholesterolemia; DM  =  diabetes mellitus; HU  =  hyperuricemia; CS  =  current smoker; Ex S  =  ex-smoker; NS  =  never-smoker; ^*^, significantly different from CS (p<0.01); ^†^, significantly different from Ex S (p<0.01).

### Relationship between the severity of airflow limitation and metabolic abnormalities in men and women without asthma

The influence of the severity of airflow limitation on metabolic disorders was analyzed between subjects with mild airflow limitation and those with moderate-to-very-severe airflow limitation (Table 4). There was no statistically significant difference in any parameter except CRP (p<0.05) in men. On the other hand, women with moderate-to-very-severe airflow limitation had higher BMI (p<0.0001), higher frequencies of hypertriglyceridemia (p<0.0001), low HDL cholesterolemia (p<0.01), high LDL cholesterolemia (p<0.05), DM (p<0.05), and CRP (p<0.05) than in those with mild airflow limitation.

**Table 4:** Relationship between the severity of airflow limitation and metabolic abnormalities in men and women with FEV_1_/FVC ratio < 0.7.

Parameter		Men		Women	
% pFEV1		≧80%	<80%	≧80%	<80%
n		645	342	151	58
BMI	(kg/m^2^)	23.6	23.7	21.8	23.7^‡^
h-TG	(%)	30.4	29.5	4.6	15.5^‡^
l-HDL	(%)	13.2	13.7	4.6	15.5^†^
h-LDL	(%)	35.2	37.1	37.1	55.2^*^
DM	(%)	14.4	18.7	5.3	15.5^*^
HU	(%)	29.2	28.5	0.0	1.7
Alb	(g/dl)	4.4	4.4	4.4	4.4
CRP	(mg/dl)	0.15	0.21^*^	0.09	0.19^*^

*Definition of abbreviations*: FEV_1_  =  forced expiratory volume in 1 second; % pFEV_1_  =  FEV_1_/predicted FEV_1_×100%; BMI  =  body mass index; h-TG  =  hypertriglycemia; l-HDL  =  low high-density lipoprotein cholesterolemia; h-LDL  =  high low-density lipoprotein cholesterolemia; DM  =  diabetes mellitus; HU  =  hyperuricemia; Alb  =  albumin; CRP  =  C-reactive protein; Data are expressed as mean or percentage; ^*^, p<0.05; ^†^, p<0.01; ^‡^, p<0.0001.

### Relationship among smoking index, length of smoking cessation, and metabolic disorders in men

To evaluate the dose-dependent effect of cigarette smoking on each parameter, a logistic regression analysis between the frequency of abnormality of each parameter and SI was performed in men. Because there was no significant difference in any parameter among current smokers, ex-smokers, and never-smokers in any age group in women, we performed this analysis only in men. Univariate regression analysis showed that SI was associated with increased frequencies of metabolic abnormalities except high LDL cholesterolemia, and that BMI was associated with increased frequencies of all metabolic abnormalities examined. After adjustment for age, BMI, and alcohol index (g/week × year), multiple logistic regression analysis revealed that SI was an independent factor associated with increased frequencies of hypertriglyceridemia (OR 1.015; 95% CI: 1.012–1.018; p<0.0001), low HDL cholesterolemia (1.013; 1.010–1.016; p<0.0001), DM (1.010; 1.007–1.013; p<0.0001), and HU (1.004; 1.001–1.007; p = 0.0041), but not high LDL cholesterolemia (Table 5). Furthermore, we evaluated the relationship between length of smoking cessation and comorbidities in male ex-smokers. Univariate regression analysis showed that among the metabolic abnormalities, the presence of hypertriglyceridemia and HU showed correlations with the length of smoking cessation. After adjusting for age, BMI, alcohol index and SI, multiple logistic regression analysis showed that the length of smoking cessation was an independent factor associated with a decrease in the frequency of hypertriglyceridemia (OR, 0.984; 95% CI: 0.975–0.994; p = 0.0007), but not HU (1.001; 0.993–1.008; p = 0.8568; Table 6).

**Table 5:** Univariate and multivariate logistic regression analysis for frequency of metabolic abnormalities in men.

		Univariate analysis			Multivariate analysis		
	Variables	OR	95% CI	P value	OR	95% CI	P value
h-TG	Age	0.993	0.988–0.998	0.0074	0.993	0.988–0.999	0.0125
	BMI	1.220	1.200–1.239	<0.0001	1.217	1.198–1.237	<0.0001
	SI	1.015	1.012–1.017	<0.0001	1.015	1.012–1.018	<0.0001
	AI	1.014	1.007–1.022	0.0002	1.008	1.001–1.016	0.0368
							
l-HDL	Age	1.018	1.011–1.025	<0.0001	1.019	1.011–1.026	<0.0001
	BMI	1.143	1.121–1.165	<0.0001	1.147	1.125–1.170	<0.0001
	SI	1.013	1.010–1.016	<0.0001	1.013	1.010–1.016	<0.0001
	AI	0.968	0.960–0.975	<0.0001	0.955	0.943–0.966	<0.0001
							
h-LDL	Age	1.009	1.004–1.014	0.0002	1.012	1.007–1.017	<0.0001
	BMI	1.113	1.097–1.129	<0.0001	1.116	1.100–1.132	<0.0001
	SI	1.002	1.000–1.004	0.0820	1.002	1.000–1.005	0.0813
	AI	0.968	0.960–0.975	<0.0001	0.966	0.959–0.974	<0.0001
							
DM	Age	1.055	1.048–1.062	<0.0001	1.057	1.050–1.065	<.0001
	BMI	1.101	1.082–1.121	<0.0001	1.125	1.104–1.147	<.0001
	SI	1.015	1.013–1.018	<0.0001	1.010	1.007–1.013	<.0001
	AI	1.004	0.994–1.014	0.4408	0.999	0.989–1.009	0.8288
							
HU	Age	0.986	0.981–0.990	<0.0001	0.988	0.983–0.993	<.0001
	BMI	1.146	1.129–1.163	<0.0001	1.142	1.125–1.160	<.0001
	SI	1.005	1.002–1.007	0.0002	1.004	1.001–1.007	0.0041
	AI	1.023	1.015–1.031	<0.0001	1.023	1.015–1.031	<.0001

*Definition of abbreviations*: OR  =  odds ratio; CI  =  confidence interval; BMI  =  body mass index; SI  =  smoking index; AI  =  alcohol index; h-TG  =  hypertriglycemia; l-HDL  =  low high-density lipoprotein cholesterolemia; h-LDL  =  high low-density lipoprotein cholesterolemia; DM  =  diabetes mellitus; HU  =  hyperuricemia.

**Table 6:** Univariate and multivariate logistic regression analysis for frequency of metabolic abnormalities in male ex-smokers.

		Univariate analysis			Multivariate analysis		
	Variables	OR	95% CI	P value	OR	95% CI	P value
h-TG	Age	0.992	0.984–1.001	0.0816	1.000	0.989–1.011	0.9801
	BMI	1.209	1.176–1.242	<0.0001	1.198	1.166–1.232	<0.0001
	SI	1.012	1.008–1.017	<0.0001	1.006	1.001–1.012	0.0321
	AI	1.010	0.998–1.023	0.1048	1.008	0.995–1.021	0.2505
	Smoking cessation (yr)	0.974	0.967–0.982	<0.0001	0.984	0.975–0.994	0.0007
							
HU	Age	0.986	0.977–0.994	0.0010	0.987	0.977–0.998	0.0159
	BMI	1.149	1.119–1.179	<0.0001	1.143	1.113–1.174	<0.0001
	SI	1.005	1.001–1.010	0.0156	1.005	1.000–1.011	0.0665
	AI	1.023	1.010–1.036	0.0003	1.022	1.009–1.035	0.0009
	Smoking cessation (yr)	0.990	0.983–0.997	0.0037	1.001	0.993–1.008	0.8568

*Definition of abbreviations*: OR  =  odds ratio; CI  =  confidence interval; BMI  =  body mass index; SI  =  smoking index; AI  =  alcohol index; h-TG  =  hypertriglycemia; HU  =  hyperuricemia.

## Discussion

We found that airflow limitation is more common in current smokers than in ex-smokers or never-smokers in both men and women. In addition, the frequency of hypertriglyceridemia is higher in current smokers in all age groups whereas low HDL cholesterolemia and DM are higher in current smokers in the ≥ 40 s age group than in ex-smokers or never-smokers in men, but not in women. Moreover, the presence of moderate-to-very-severe airflow limitation is associated with higher BMI, and higher frequencies of hypertriglyceridemia, low HDL cholesterolemia, high LDL cholesterolemia, and DM compared to mild airflow limitation in women, but not in men. Finally, SI is an independent factor associated with an increased frequency of hypertriglyceridemia, low HDL cholesterolemia, DM, and HU in men, and the length of smoking cessation is an independent factor associated with a decreased frequency of hypertriglyceridemia.

The prevalence of airflow limitation in the present study was 9.2% in men and 3.1% in women (6.9% overall), which is lower than in a previous study (the NICE study) in Japan [2] which reported 16.4% in men, 5.0% in women (10.9% overall). The differences may be explained by differences in the demographics of the subjects, especially in age and occupation; the ages were > 30 in our study and > 40 in the NICE study. The occupation was mostly school teachers in our study; on the other hand, various occupations were included in the NICE study. In the NICE study, only 9.4% of cases with airflow limitation reported previous diagnoses of COPD [2]; thus, under-recognition of COPD is a serious problem. The UPLIFT trial and subgroup analysis [17], [18] showed that early intervention with tiotropium reduced the rate of decline in FEV_1_ and improved health status, time to first exacerbation, and time to exacerbation resulting in hospital admission in subjects with GOLD stage II COPD. These studies revealed that early diagnosis and appropriate intervention for COPD is very important. In Japan, medical checkups are conducted annually at most work sites. However, spirometry is not readily available, and thus is rarely performed. In the United States, the National Lung Health Education Program has been created with a mission of increasing COPD awareness and for education, and spirometry is recommended as a screening tool to detect airflow limitation during its early stages [19], [20]. On the other hand, the GOLD 2011 COPD guidelines advocate active case finding but not screening spirometry [21], [22]. Thus there is no consensus about the use of spirometry as a screening tool. Nonetheless, we believe that spirometry is needed at every institution providing medical checkups to detect airflow limitation in the preclinical stage from the standpoint of preventative medicine.

Various mechanisms of smoking-induced changes in lipid levels in blood have been demonstrated. Nicotine stimulates the sympathetic adrenal system, resulting in the increased secretion of catecholamines that results in increased lipolysis and increased concentrations of plasma free fatty acids, which further promote the secretion of hepatic free fatty acids and TG, along with VLDL-C, into the blood stream [23]. In chronic smokers, lipoprotein lipase activity decreases in skeletal muscle and adipose tissue, resulting in delayed metabolism of TG-rich lipoproteins [24], [25]. The relationship between airflow limitation and metabolic profile is also becoming clearer, but there is still some controversy. Most previous studies have shown that subjects with airflow limitation have a higher frequency of hypertriglyceridemia [26], [27], high LDL cholesterolemia [26], [28]–[30], low HDL cholesterolemia [26], [28]–[30], and DM [26], [27]. In contrast, a few studies have reported no significant differences in the levels of HDL-C [27], [31], [32], LDL-C [31], [32], or TG [32] between subjects with and without airflow limitation.

We found that male smokers had high frequencies of hypertriglyceridemia, low HDL cholesterolemia, and DM compared to never-smokers and ex-smokers, consistent with the findings of previous studies [10], [11]. In contrast, no such difference was found in women. We also found that women with moderate-to-very-severe airflow limitation had higher BMI and higher frequencies of hypertriglyceridemia, low HDL cholesterolemia, high LDL cholesterolemia, and DM than those with mild airflow limitation, but no such difference was found in men. These results indicate gender differences in the relationships or interactions among tobacco smoking, airflow limitation, and metabolic abnormalities. One previous study [33] reported that women may actually be at greater risk of smoking-induced impairment of lung function, more severe dyspnea, and poorer health status with the same levels of tobacco exposure. Another previous study [34] showed that female patients with COPD presented more frequently with chronic heart failure, osteoporosis, and DM. There is emerging evidence that women may be biologically more susceptible to the harmful effects of cigarette smoke than men [27], [35]. In an animal model, sex differences in the metabolism of cigarette smoke have been demonstrated [36]. These differences were mediated by cytochrome P450 (CYP) and resulted in increased production of toxic and carcinogenic airway molecules in women. Although the mechanism remains unclear, estrogen can upregulate CYP enzyme [37]. Further research focusing on gender differences in susceptibility to cigarette smoke is required.

In the present study, we also found that SI was an independent factor associated with an increase in the frequency of hypertriglyceridemia, low HDL cholesterolemia, DM, and HU. Smoking may cause increases in LDL-C, TG, and VLDL-C, and a decrease in HDL-C [38], [39], and DM may be more frequent in, and impact the prognosis of, COPD [5]. Our results are consistent with those reports. We also found that the length of smoking cessation was an independent factor associated with a decrease in the frequency of hypertriglyceridemia, but not low HDL cholesterolemia. A previous meta-analysis showed that smoking cessation resulted in an increase in HDL-C levels [10], but not in TG or LDL-C levels. Because the level of TG has been shown to be an independent risk factor for stroke or cardiovascular diseases [40]–[42], early intervention for smoking cessation will have an additional effect on lipid metabolism and be helpful in preventing fatal cerebro- and cardiovascular diseases.

Our study has some limitations. First, the subjects were school workers, mostly teachers. The National Survey in Japan reported that 39.4% of men (55.6% in their 30 s, 49.1% in 40 s, 42.3% in 50 s, and 32.8% in 60 s) and 11.0% of women (17.2% in 30 s, 17.9% in 40 s, 9.3% in 50 s, and 7.3% in 60 s) were current smokers [43], suggesting that school teachers smoke far less than many other workers. This could be because school teachers are generally more diligent and strict about lifestyle-related habits. Thus, this bias should be taken into account when considering the nutritional data in the present study. Second, this was a cross-sectional study, and the subjects were not tracked over time. A future prospective study, especially on the relationship between smoking cessation and pulmonary function, would be useful. Third, we have no data regarding diet or excise in the subjects. We also have no air pollution data for the daily living areas of the subjects. Those factors may be confounding factors for frequencies of airflow limitation and metabolic abnormalities. Dietary intake patterns have been associated with the development of COPD and airflow limitation [44], and air pollution is a major risk factor not only for COPD and cardiovascular disease [45],[46] but also for elevated blood lipid levels [47], [48], diabetes, and metabolic syndrome [49]. Fourth, as post-bronchodilator spirometry was not performed in our study, the diagnosis of COPD was not made strictly according to the GOLD guidelines; our data only indicate relationships between metabolic abnormalities and airflow limitation, but not COPD. Finally, we excluded physician-diagnosed asthmatic patients in the analysis of frequency of airflow limitation, but the possibility that there were still undiagnosed asthmatics cannot be ruled out.

A strength of our study is that it was conducted at a single large institution, and the data obtained are expected to show less variability than those from multi-institutional studies because all of the data were obtained under the same conditions. The subjects stayed at the same hospital and had the same pretest dinner, which may minimize the influence of diet among subjects just before the examination day. Also, the blood drawing and pulmonary function tests were performed at a scheduled time.

## Conclusions

Smoking habits cause high incidences of metabolic abnormalities including hypertriglyceridemia, low HDL cholesterolemia, DM, and HU in men, but not in women. Women, but not men, with airflow limitation had higher frequencies of metabolic abnormalities. Smoking index was an independent factor associated with increased frequencies of those metabolic abnormalities in men, and smoking cessation was associated with a decrease in the frequency of hypertriglyceridemia.
